# Awareness, knowledge and practices related to intra-abdominal hypertension and abdominal compartment syndrome among intensive care providers: a systematic scoping review

**DOI:** 10.1186/s13613-025-01521-4

**Published:** 2025-07-24

**Authors:** ZhiRu Li, FangYan Lu, JingYun Wu, YanHong Dai, Yan Wang, Li Zheng, HuaFen Wang

**Affiliations:** 1 Department of Nursing, The First Affiliated Hospital, Zhejiang University School of Medicine, 79 Qingchun Road, Hangzhou, Zhejiang China; 2 Department of Nursing, Zhejiang University School of Medicine, Hangzhou, Zhejiang China; 3 Hepatobiliary and Pancreatic Surgery, The First Affiliated Hospital, Zhejiang University School of Medicine, Hangzhou, Zhejiang China

**Keywords:** Intra-abdominal pressure, Intra-abdominal hypertension, Abdominal compartment syndrome, Intensive care, Scoping review

## Abstract

**Objectives:**

To provide a comprehensive overview of current research on intensive care providers’ awareness, knowledge, and practices regarding IAP/IAH/ACS, as well as barriers to IAP measurement.

**Methods:**

This scoping review was guided by the framework of Arksey and Malley. Eight databases were searched to identify research published after 2007, including MEDLINE Complete, EMBASE, Web of Science, Cochrane Library, CINAHL Complete, ProQuest Health & Medical Complete, CNKI, and WANFANG. Two researchers reviewed and screened potentially relevant studies based on title and abstract. Full-text articles were independently assessed for eligibility based on predefined inclusion criteria.

**Results:**

Nineteen articles were included. Overall, pediatric intensive care providers demonstrated a lower awareness and knowledge of IAH/ACS compared to adult intensive care providers, particularly regarding the consensus definitions of IAH/ACS in critically ill children. IAP measurement has not been adequately integrated into clinical practice, with 18.0–73.0% of intensive care providers reporting they have never measured it. The frequency of IAP measurements and the criteria for determining which patients necessitate such measurements exhibited significant variability across different hospitals. The most frequently mentioned barriers to IAP measurement include a lack of knowledge regarding IAP measurement among adult intensivists, an overreliance on physical examination among pediatric intensivists, uncertainty in interpreting IAP data among adult intensive care nurses, and challenges in identifying populations at high risk of IAH among pediatric intensive care nurses. Diuretics were mentioned most often in the management of IAH/ACS, followed by administration of vasopressors and inotropes, decompressive laparotomy, and judicious administration of fluids and blood products. 37.0–66.3% of adult intensivists would choose a decompressive laparotomy in cases of ACS, whereas pediatric intensivists were less inclined to opt for the same approach.

**Conclusions:**

Since the publication of the WSACS consensus in 2007, there has been an improvement in awareness and knowledge regarding IAP/IAH/ACS among intensive care providers. Nevertheless, the understanding of the consensus definitions regarding IAH/ACS remains inadequate, particularly among pediatric intensive care providers. It is imperative to advocate for the implementation of WSACS guidelines in hospitals through targeted training programs and to promote the routine practice of IAP measurement in clinical settings.

## Introduction

Intra-abdominal hypertension (IAH) and abdominal compartment syndrome (ACS) are increasingly recognized as potentially life-threatening complications that significantly contribute to multiple organ failure and are associated with considerable morbidity and mortality [1–3]. An estimated 50.0–80.0% of critically ill adults are at risk of developing IAH, with a progression to ACS occurring in 2.7–51.7% of cases, depending on the specific patient population [4, 5]. ACS is also prevalent among critically ill pediatric patients, with an incidence ranging from 3.3 to 63.0%, and a mortality rate that can reach as high as 40.0–60.0% [6, 7]. Such conditions lead to increased resource utilization and pose a significant economic burden on healthcare systems [4, 8]. Consequently, it is imperative for intensive care providers to be adept at recognizing, managing, and, most importantly, preventing ACS in high-risk populations, in order to reduce its associated morbidity and mortality.

To promote research, education, awareness, and standardized management of IAH and ACS, the World Society of the Abdominal Compartment Syndrome (WSACS; www.wsacs.org) was founded in 2004 by an international group of clinicians. The WSACS subsequently published consensus definitions and recommendations pertaining to IAH/ACS in 2006 and 2007 [9, 10]. In 2013, the WSACS updated guidelines extended specific considerations for pediatric patients and new concepts, and evaluated the treatment strategy based on the GRADE (Grading of Recommendations, Assessment, Development, and Evaluation) system [11]. Nevertheless, a subsequent international survey conducted after the updated guidelines revealed that awareness, knowledge, and application of evidence-based medicine regarding IAH/ACS among adult intensive care providers were considered insufficient [12]. Furthermore, data from self-reported surveys indicate that the incorporation of intra-abdominal pressure (IAP) measurement into clinical practice remains inadequate [12], even in situations where evidence suggests it may influence patient management and outcomes [13, 14].

Multiple studies have been conducted to identify the current state of awareness, knowledge, and use of evidence-based practices regarding IAP/IAH/ACS among intensive care providers [15, 16]. However, it remains unclear to what extent intensive care providers are aware of the definitions and recommendations proposed by the WSACS, their perceived importance, and their application in the clinical management of IAH/ACS. Therefore, this study aimed to systematically review current research on intensive care providers’ knowledge, perceptions, and practices regarding IAP, IAH, and ACS.

## Methods

### Design

Given the broad scope of the research question, a scoping review was chosen as an appropriate approach for our study [17]. This approach facilitates the collection of information from different sources and the design of studies addressing diverse research questions. We structured the stages of our scoping review using the five-step methodological framework proposed by Arksey and Malley [18]. The Preferred Reporting Items for Systematic Reviews and Meta-Analysis extension for Scoping Reviews (PRISMA-ScR) checklist was followed to promote study quality and rigor [19].

### Stage 1: identifying the research question

Two specific questions that we used to guide this review were as follows: (1) What are intensive care providers’ awareness, knowledge, and practices related to IAH and ACS? and (2) What is the current status and potential barriers to the implementation of IAP measurements?

### Stage 2: identifying the relevant studies

Eight electronic databases, including six English databases (MEDLINE Complete, EMBASE, Web of Science, Cochrane Library, CINAHL Complete, and ProQuest Health & Medical Complete) and two Chinese databases (CNKI and WANFANG), were systematically searched for articles published after 2007. The search strategy was developed in consultation with an information scientist, and the following keywords were used: (intra-abdominal pressure OR intra-abdominal hypertension OR abdominal compartment syndrome) AND (critical care OR intensive care) AND (knowledge OR awareness OR attitude OR practice OR perception OR behavior OR barrier OR obstacle OR facilitator). Publications in both Chinese and English were retrieved, and translations of Chinese literature were independently verified by two bilingual authors. The reference lists of included studies were reviewed to identify additional relevant studies.

### Stage 3: determining the study selection

After removing duplicates, all retrieved articles were independently screened by the same two researchers in two stages: title/abstract screening and full-text screening. Each article was labeled as ‘include’, ‘exclude’ or ‘unclear’ based on title and abstract. Afterwards, studies scored as ‘include’ or ‘unclear’ were acquired in full text, and two researchers independently reviewed studies to decide whether to include them. Any discrepancies during the screening process were discussed and resolved with a third researcher. Study selection was based on inclusion and exclusion criteria. The inclusion criteria were (1) original studies to identify knowledge, awareness, perceptions, attitudes, practices, or skills related to IAP, IAH and ACS among intensive care providers; (2) barriers or facilitators of IAP measurement; (3) full-text articles; and (4) studies published after 2007 in peer-reviewed journals. The date restriction was made to better understand the gap between evidence and clinical practice of IAP, IAH, and ACS after the report of the updated consensus in 2007. The exclusion criteria were (1) studies focused on the experience of intensive care providers in IAP, IAH, and ACS management and (2) conference abstracts, reviews, study protocols, and duplicate publications.

### Stage 4: charting the data

Each identified article was carefully assessed by the first author and cross-checked by another reviewer to ensure accuracy. A standardized table was used to extract the following data from each study: the first author, publication year, country, research design, research purpose, participants, sample size and main findings. The quality of included studies was assessed based on the transparency, rigor of method and production of results (e.g. detailed descriptions of study design, sample selection, measurement methods; the method meets the aim, the inclusion and exclusion criteria are reasonable, the measurement tools are reliable), as the PRISMA guidelines do not require the use of critical appraisal tools. The final extracted data were reviewed and discussed by the research team.

### Stage 5: collating, summarizing, and reporting the data

Two independent researchers extracted data relevant to the review questions and compared methodologies, samples, measurements, and results. We used descriptive tables to synthesize and compare a wide range of findings, including specific details about the knowledge, perceptions, practices, and potential barriers of IAP, IAH and ACS among intensive care providers. Research team members jointly participated in the discussion and revision of findings, and they also assisted in verifying the identification and description of themes throughout the process.

## Results

**Fig. 1:**
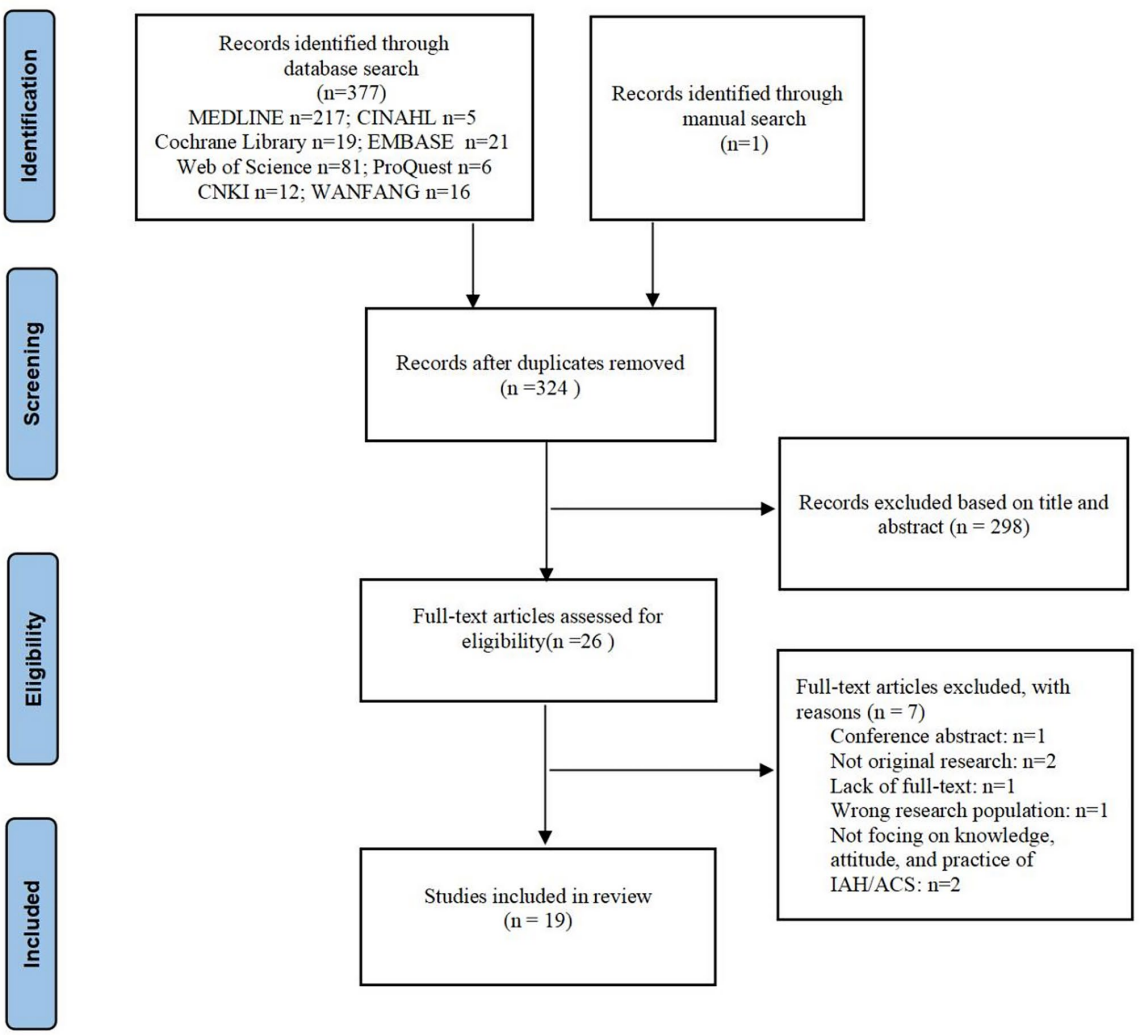
PRISMA flow diagram

We identified 377 articles from eight electronic databases and one additional article through other sources. In total, there were 378 studies, from which 54 duplicates were removed. A total of 324 records were screened based on article titles and abstracts. From these, 298 articles were excluded, resulting in 26 articles being evaluated for inclusion. After reviewing the remaining full-text articles, 7 were excluded for the following reasons: conference abstract (*n* = 1), unavailable full-text (*n* = 1), not original research (*n* = 2), wrong research population (*n* = 1), and lack of focus on knowledge, attitudes, and practices related to IAH/ACS (*n* = 2). Ultimately, 19 articles were included in this review [12, 15, 16, 20–35]. A PRISMA flow chart is displayed in Fig. 1.

### Characteristics of the included studies

**Fig. 2:**
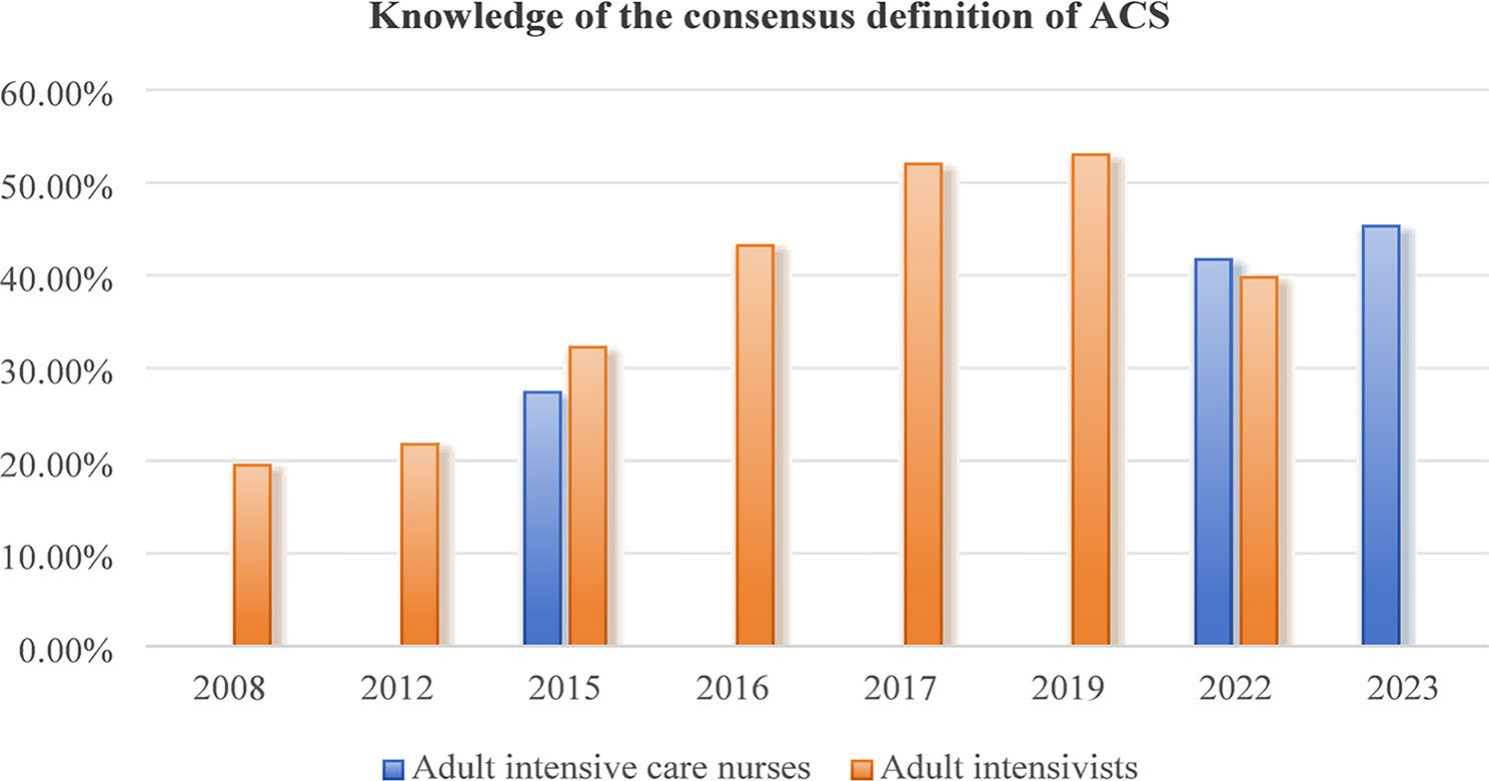
Knowledge of the consensus definition of ACS among adult intensivists and adult intensive care nurses

**Fig. 3:**
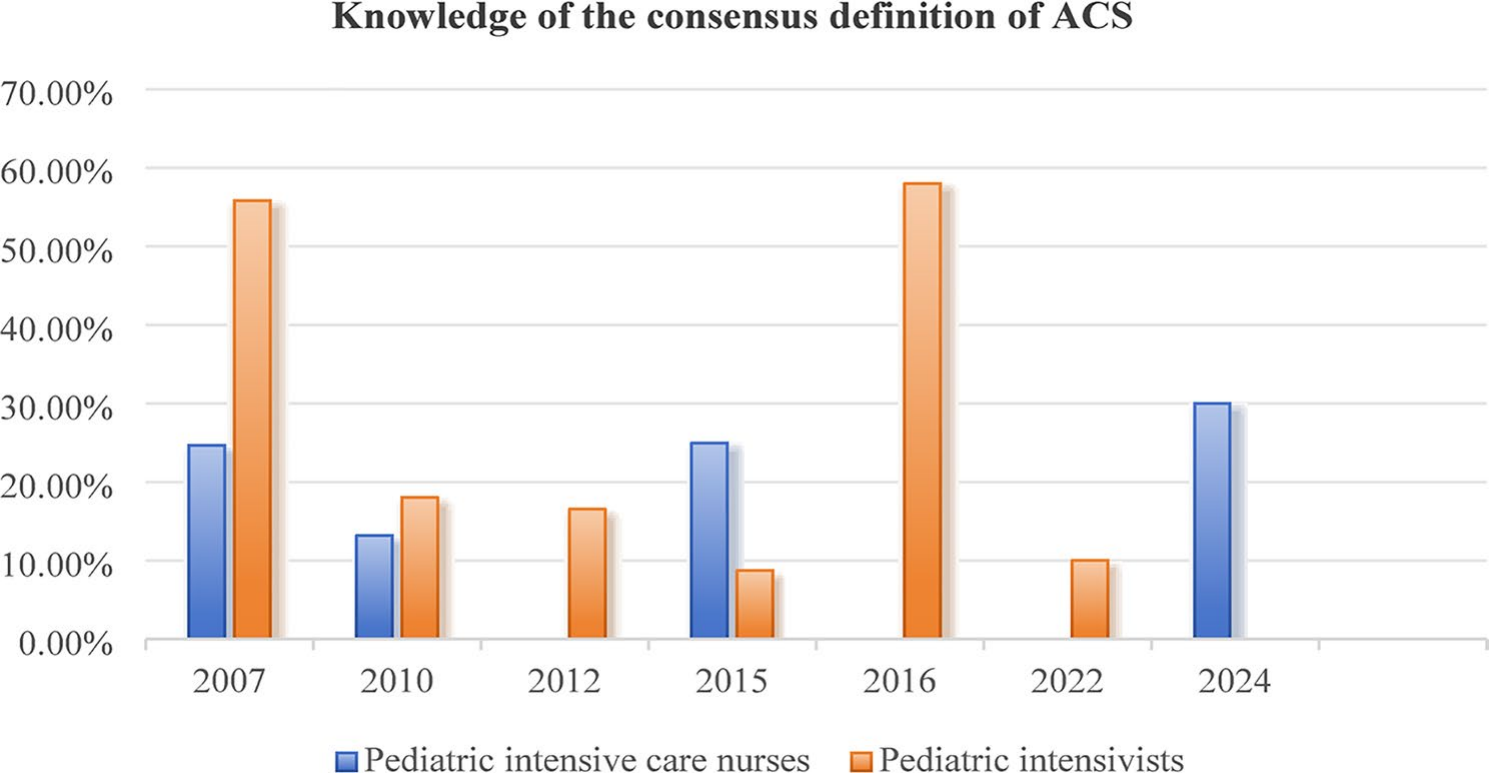
Knowledge of the consensus definition of ACS among pediatric intensivists and pediatric intensive care nurses

**Fig. 4:**
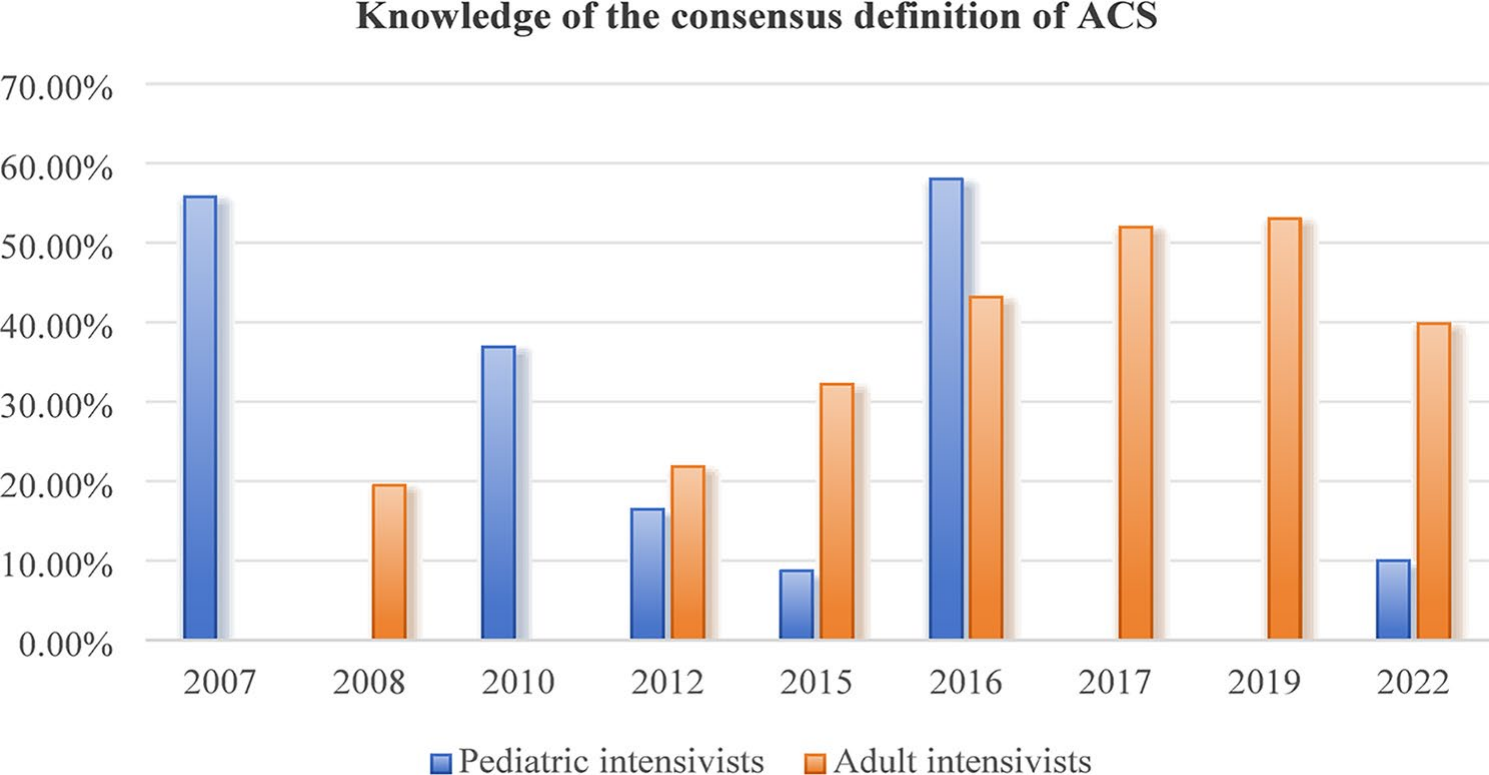
Knowledge of the consensus definition of ACS among pediatric intensivists and adult intensivists

**Fig. 5:**
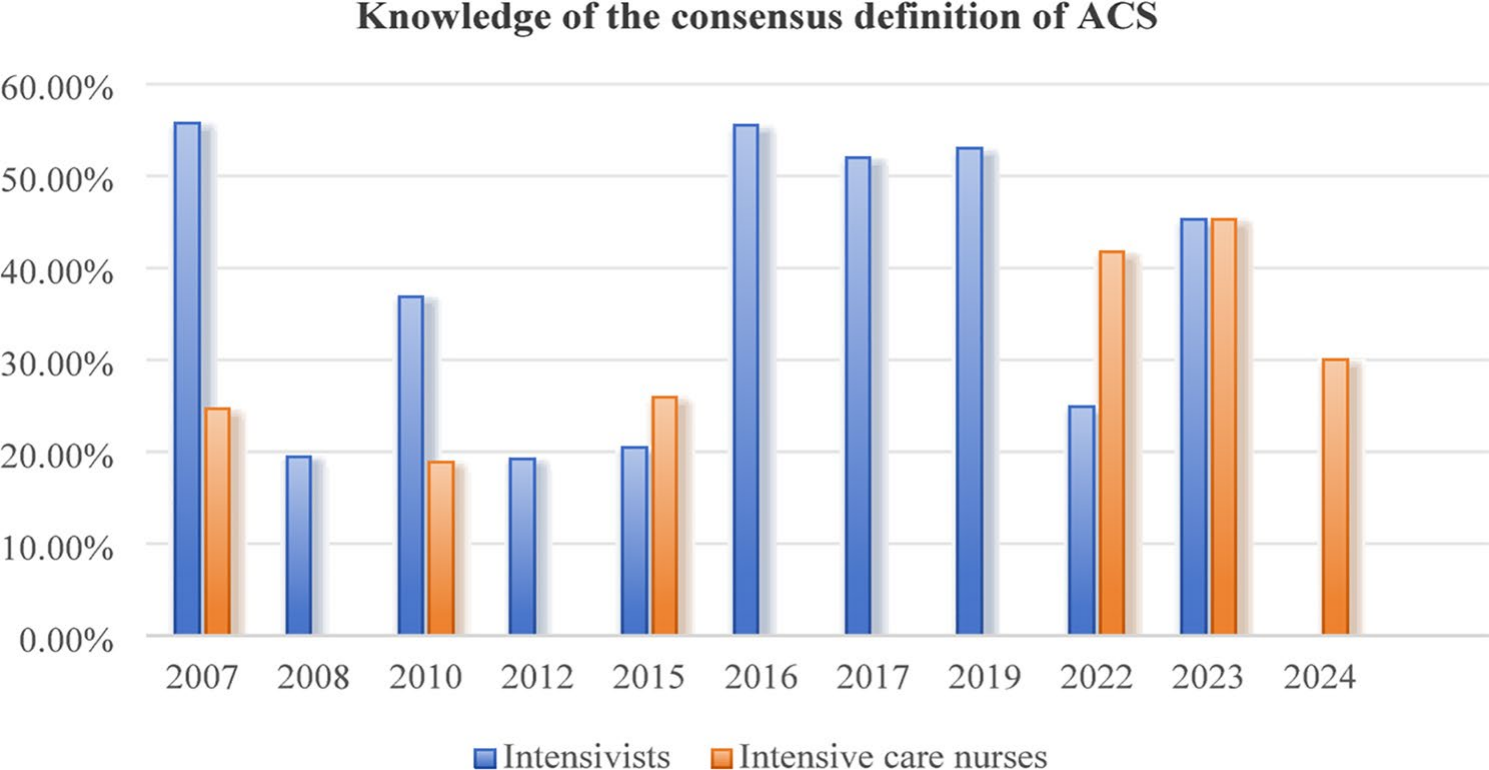
Knowledge of the consensus definition of ACS among intensivists and intensive care nurses

**Fig. 6:**
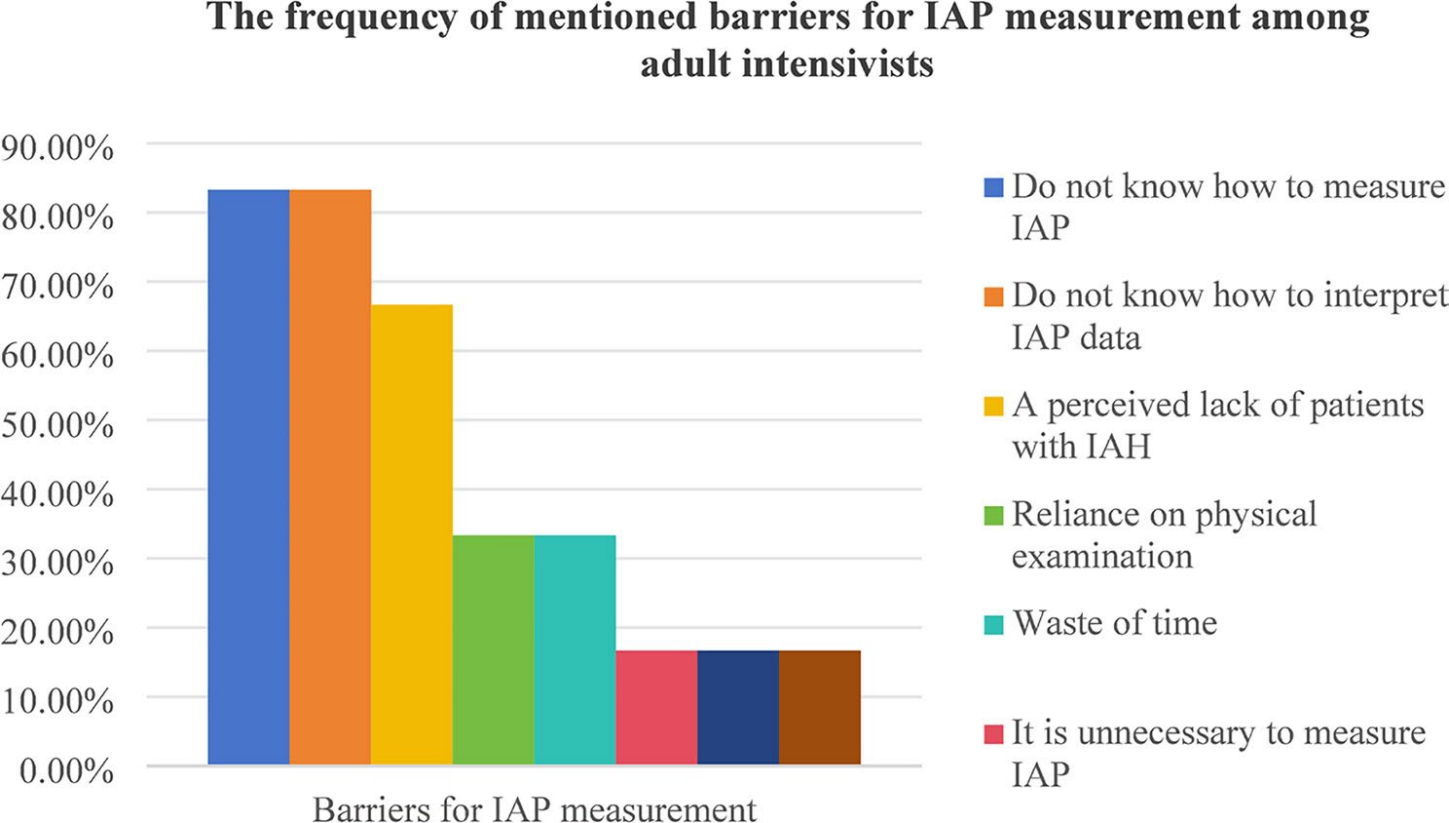
The frequency of mentioned barriers for IAP measurement among adult intensivists

**Fig. 7:**
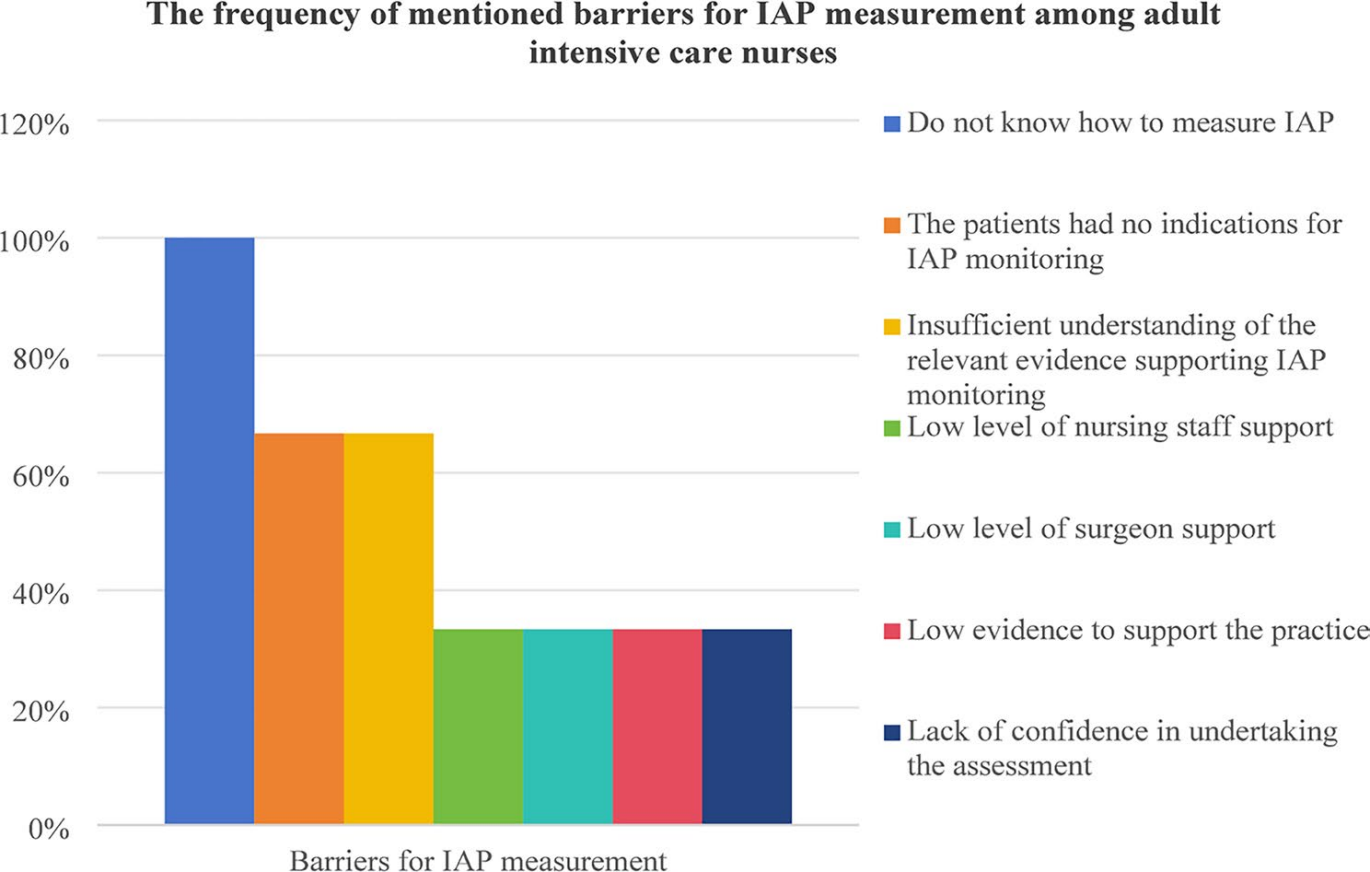
The frequency of mentioned barriers for IAP measurement among adult intensive care nurses

**Fig. 8:**
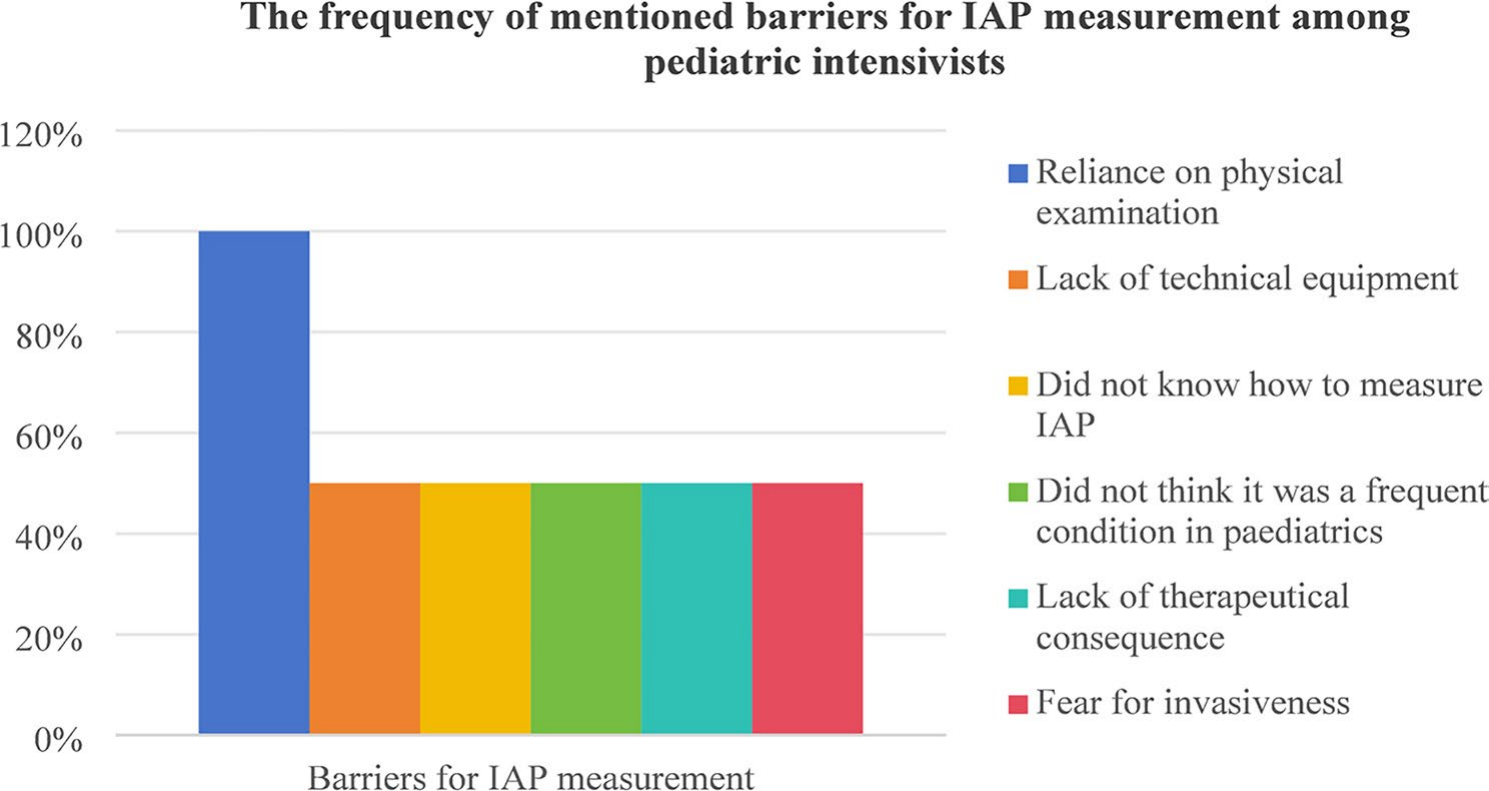
The frequency of mentioned barriers for IAP measurement among pediatric intensivists

**Fig. 9:**
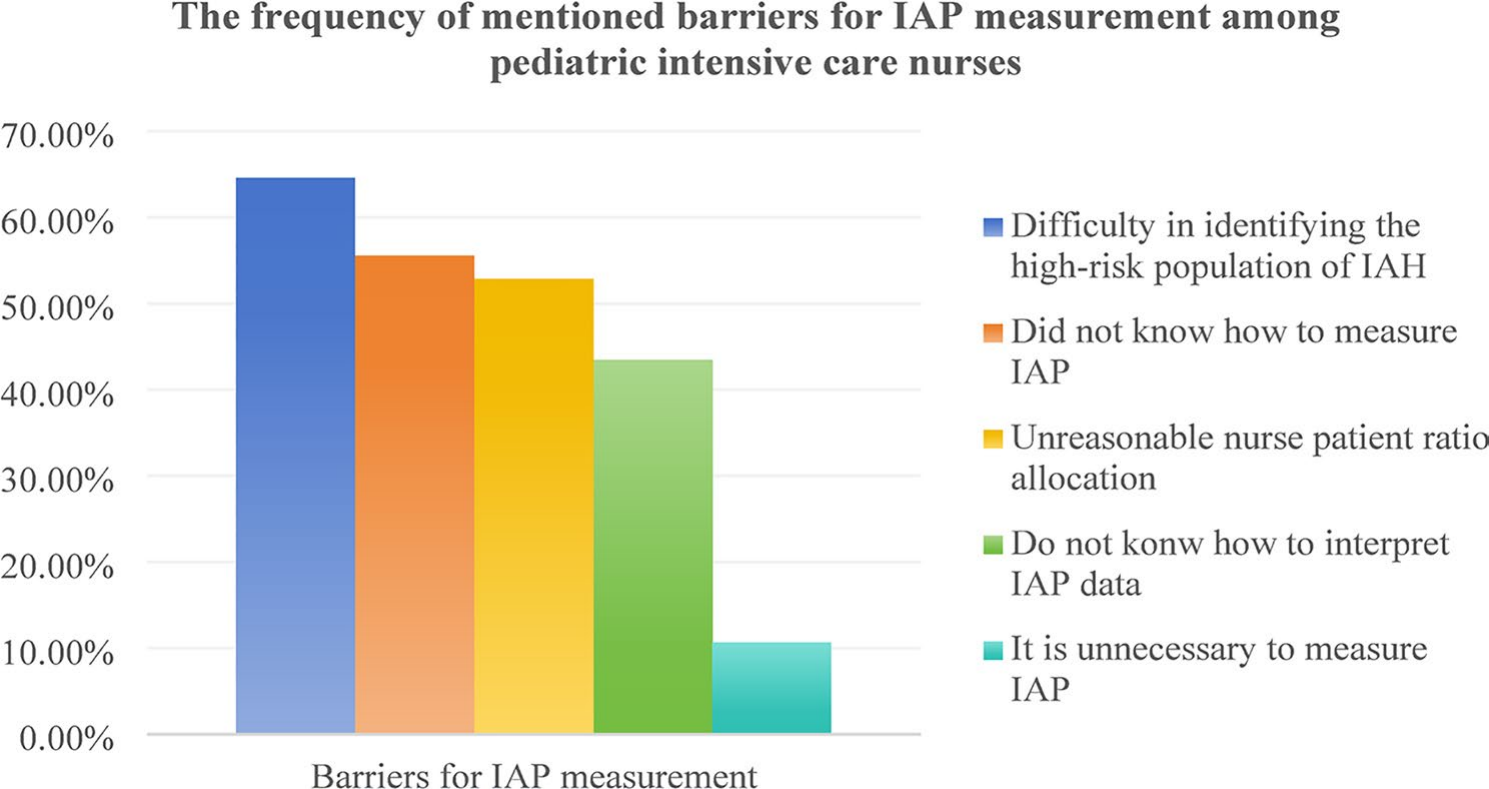
The frequency of mentioned barriers for IAP measurement among pediatric intensive care nurses

Table 1 summarizes the main characteristics of each included study. Nineteen articles were identified as concentrating on evaluating the state of awareness, knowledge, and practice regarding IAP/IAH/ACS among intensive care providers (Figs. 2, 3, 4, 5, 6, 7, 8, and 9). These studies were conducted in different countries and regions, including Africa (*n* = 3) [12, 16, 26], China (*n* = 6) [20–22, 28, 29, 33], the USA (*n* = 2) [31, 34], Germany (*n* = 2) [23, 30] and Saudi Arabia (*n* = 2) [24, 25], with one study each from Australia [15], Italy [35], the Netherlands [27], and Brazil [32]. The year of publication ranged from 2008 to 2024. All included studies reported using quantitative methods, with a cross-sectional study design. The studies primarily involved healthcare providers working in various intensive care units, including emergency, cardiac, surgical, medical, neonatal, and pediatric ICUs. A total of 7363 participants were included in this review. The respondents primarily belonged to the professions of intensivists (37.2%, 2742) and intensive care nurses (41.9%, 3084). Among them, 11.0% were pediatric intensivists, 16.8% were pediatric intensive care nurses, 36.1% were adult intensive care nurses, and 36.0% were adult intensivists. The sample size in each study ranged from 32 to 2244 participants. Data from the 19 studies were analyzed and categorized into four main themes: awareness of IAH/ACS, knowledge of IAH/ACS, IAP measurement practices, and treatments for IAH/ACS.

**Table 1:** Basic characteristics of included studies (*N* = 19)

Study	Country	Method	Aim	Sampling	Main findings
**Pediatric intensive care nurses**					
Li et al. 2024 [20]	China	A cross-sectional survey. Method: an online survey using a self-made questionnaire with 34 questions	To assess the knowledge, attitudes and practices of pediatric intensive care nurses regarding IAP monitoring in China	A convenience sample of 212 pediatric intensive care nurses in eight hospitals in China	Knowledge: The knowledge score was 7.84 ± 2.35, with a scoring rate of 56.0%; only 21.5% of nurses were aware of the correct perfusion volume for intravesical pressure measurement in children, only 30.0% were aware of the definition of IAH/ACS in children, and only 48.3% were aware of the risk factors for IAH; nurses who have received IAP monitoring training has better KAP scores than those who have not received (*P* < 0.05). Practice: the practice score was 28.44 ± 6.46, with a scoring rate of 69.3%; 119 (56.1%) had never received training in IAP measurement; most of nurses (81.9%) measure IAP when children have any risk factors for IAH/ACS. Barriers for IAP measurement: difficulty in identifying the high-risk population of IAH (64.6%), unfamiliar with the operation process of IAP measurement (55.6%), unreasonable nurse patient ratio allocation (52.8%), do not konw how to interpret IAP data (43.4%), it is unnecessary to measure IAP (10.7%).
Liang et al. 2015 [29]	China	A cross-sectional study Method: using a self-made questionnaire with 16 items	To investigate the knowledge of ACS and IAP measurement among Paediatric healthcare providers in china	A convenience sample of 194 physicians (84.5%) and nurses (15.5%) in neonatal and pediatric intensive care units	Awareness: 28.9%(56/194) of respondents did not heard of ACS, 50.0% of nurses heard of ACS, but did not contact ACS; with the increase of ICU working time, the proportion of medical staff aware of ACS is also increasing. Knowledge: only 25.0% (1/30) of nurses knew the correct definition of ACS; Only 52.3% (101/194) of all respondents know the correct method for IAP measuring through bladder, and only 57.1% (20/35) knew the correct saline volume; Practice: IAP was measured most frequently via the bladder (83.3%); 42.8% of respondents monitored IAP in all children with abdominal distension, while 23.8% monitored IAP in all children with acute abdomen during the perioperative period
Newcombe et al. 2012 [31]	USA	A cross-sectional study Method: using a self-made questionnaire with 10 items	To assess and compare the awareness and knowledge of ACS among pediatric critical care nurses in 2006 and 2010	A convenience sample of 433 pediatric critical care nurses participanting the national pediatric critical care nursing conferences in 2006 and 2010	Awareness: The percentage of PCCN aware of ACS was 87.8% in 2010, and “years in practice” was significantly associated with awareness of ACS. Knowledge: The understanding of the definition of ACS among PCCN still appears to be unclear as in 2010, only 13.2% of respondents correctly defined ACS in children. Practice: Almost a quarter of the nurses indicated that they had not measured IAP during management or care of their patient with ACS in 2010; the most common method of measuring IAP remains the bladder method and the percentage of nurses having direct experience using it almost doubled in 2010 (39.1%); the intra-esophageal (gastric) method is not being used in pediatric management of ACS; there is a drop in the sole use of clinical examination to monitor IAP compared to more objective methods Management: Hands-on experience with management of ACS improved from 49.1% in 2006 to 67.9% in 2010 (*p* < 0.001)
Ejike et al. 2010 [34]	USA	A cross-sectional study Method: using a self-made questionnaire with 10 items	To assess awareness of ACS, knowledge of the definition and IAP measurement techniques used among pediatric healthcare providers	A convenience sample of 517 pediatric healthcare providers participanting the national pediatric critical care nursing conference in 2006 and the world congress of pediatric critical care in 2007	Awareness: 95.0% of pediatric intensive care nurses did heard of ACS in the survey. Knowledge: only 24.7% of pediatric intensive care nurses knew the correct definition of ACS.
**Pediatric intensivists**					
Wiegandt et al. 2023 [23]	Germany	A cross-sectional study Method: using a self-made questionnaire with 12 items	To investigate the awareness, diagnostics, and therapy of IAH and ACS among healthcare providers in German-speaking pediatric hospitals	A convenience sample of 156 physicians in neonatal and pediatric intensive care units	Awareness: The majority of all ICUs stated that IAH and ACS are present in everyday clinical practice; more physicians (44% vs. 55%) reported that IAH and ACS play a role in clinical practice compared to 2010, ICUs diagnosing at least one case of ACS increased from 25 to 35%. Knowledge: The awareness of IAH/ACS among neonatal/pediatric intensivists is insufficient, especially items related to the definition and diagnosed of IAH/ACS. only 18% in 2010 and 58% in 2016 knew the correct definition of IAH/ACS. The number of ICUs that knew how to defne an ACS in accordance with the updated WSACS guidelines increased from 18% in 2010 to 58% in 2016. Practice: The number of clinics In germany measuring IAP almost doubled in 2016 compared to 2010 (20% vs. 43%%, *p* < 0,001); more than half of the respondents did not measure IAP (43%, 65/152); the majority of those measuring IAP seldom measured IAP (29%), only 3% of respondents measured IAP regularly. Management: 36% respondents using decompressive laparotomies (DLs) to reduce IAP; respondents considered that the survival rate of ACS was higher if the patient was treated surgically.
Rezeni et al. 2022 [25]	Saudi Arabia	A cross-sectional study, Method: an online survey using a self-made questionnaire with 31 questions	To obtain information regarding awareness of ACS and IAH, recognition criteria, monitoring of IAP, and experience in managing ACS among paediatric intensivists	A convenience sample of 79 physicians working in paediatric intensive care units in Saudi Arabia	Awareness: 89.9%(71/79) of the PICUs’ physicians were aware of IAH/ACS or the effect of elevated IAP on organ function, and 93.7% (*n* = 74/79) were aware of ACS Knowledge: Physicians in PICUs generally have a low awareness of the IAH/ACS, eapecially in the areas of definition of IAH/ACS, the overall mean knowledge score was 34.3 ± 20.9%. Only 10% Physicians in PICUs correctly defined ACS as per the WSACS consensus definition. Practice: A considerable proportion of respondents (32%) never measure IAP, while IAP monitoring was performed every 6 h by 28% of respondents, and every 12 h by 28% of respondents, while 12% monitored IAP when clinically indicated, and none continuously monitored the IAP; IAP was measured most frequently via the bladder (96.0%), among those only 14 (29.8%) instilled 1mL/kg in the bladder for the measurement of IAP Management: Diuresis and sedationanalgesia were the most frequently used therapeutic approaches to treat IAH and ACS, while decompression laparotomy was the less likely reported therapeutic option. Barriers for IAP measurement: relied on their physical assessment (*n* = 14, 45%), did not know how to measure IAP (*n* = 10, 32%), did not think it was a frequent condition in paediatrics (*n* = 7, 22.5%).
Liang et al. 2015 [29]	China	A cross-sectional study Method: using a self-made questionnaire with 16 items	To investigate the knowledge of ACS and IAP measurement among Paediatric healthcare providers in china	A convenience sample of 194 physicians (84.5%) and nurses (15.5%) in neonatal and pediatric intensive care units	Awareness: 74.7% of pediatric intensivists heard of ACS. Knowledge: only 8.7% of pediatric intensivists knew the correct definition of ACS. Practice: IAP was measured most frequently via the bladder (83.3%); 42.8% of respondents monitored IAP in all children with abdominal distension, while 23.8% monitored IAP in all children with acute abdomen during the perioperative period
Kaussen et al. 2012 [30]	Germany	A cross-sectional study Method: using a self-made questionnaire with 16 items	To investigate the recognition and knowledge of IAH/ACS among German pediatric intensivists	A convenience sample of 127 German pediatric intensivists	Awareness: almost half of all ICUs that responded (56/123) stated that IAH and ACS are present in everyday clinical practice, at least one case of IAH was noted by 36% (44/124) and at least one case of ACS, by 26% (32/124). Knowledge: the knowledge of definitions and guidelines regarding the diagnosis and management of IAH/ACS are unsufficient in german pediatric intensivists; no more than 3.9% (5/127) of respondents knew correct IAH diagnosis criteria, while no more than 16.5% (21/ 127) knew correct diagnosis criteria of ACS. Practice: only 20% of respondents performed routine measurements of IAP; bladder pressure was used most frequently (96%) to assess IAP; some respondents (17%) only measured IAP in cases of organ dysfunction and failure. Management: unlike older children, the likelihood of young pediatric patients undergoing more invasive therapy options earlier appears to be inversely related to their age; more invasive therapy options will not being used until IAP ≥ 20 mmHg as long as organ dysfunction remains absent. Barriers for IAP measurement: clinical diagnosis (IAP measurement not necessary) (48%), lack of technical equipment (42%), lack of therapeutical consequence (11%), fear for invasiveness (9%); Facilitators to IAP measurement: the procedure and technical requirements became easier and more standardized
Ejike et al. 2010 [34]	USA	A cross-sectional study Method: using a self-made questionnaire with 10 items	To assess awareness of ACS, knowledge of the definition and IAP measurement techniques used among pediatric healthcare providers	A convenience sample of 517 pediatric healthcare providers participanting the national pediatric critical care nursing conference in 2006 and the world congress of pediatric critical care in 2007	Awareness: 77.8% (399/513) of participants had heard of ACS, 89.9%(71/79) of the pediatric intensivists were aware of IAH/ACS, and those working in ICUs demonstrated a greater awareness of ACS(OR = 2.9, 95%CI (1.60, 5.37). Knowledge: 55.8% of pediatric intensivists claimed to know the correct definition of ACS. Practice: 24.2% (83/343) indicated that they had never measured IAP; pediatric intensivists were more likely to measure IAP 44.9% of the time than nurses and 64.9% more often than other subspecialties; the method used most commonly was the intravesical technique (210/311,67.5%), while clinical exams alone were used by 20.3% (63/311) to detect IAH. Management: 66% (264/399) of pediatric healthcare providers indicated personal experience in management of a child with ACS
**Adult intensivists**					
Qutob et al. 2022 [24]	Saudi Arabia	A Cross-Sectional Study Method: an on-line survey using the an adapted version of the 52-item questionnaire originally developed by Wise et al.	To determine the knowledge and management of ACS and IAH among physicians in Saudi Arabia.	A convenience sample of 266 physicians in intensive care unit and non-intensive care unit	Awareness:73.7% said they were familiar with ACS; around onethird (28.2%) of them reported that they have seen 1 to 5 ACS cases in the last year, while 38.0% of them did not monitor ACS. Knowledge: Only 33.8% knew the correct definition of IAH, while 39.8% knew the correct definition of ACS. Physicians demonstrated a low level of IAP and ACS knowledge with a median score of 3.00 (IQR: 5.00–2.00), which represents 27.3% of the maximum attainable score; the median knowledge score showed a significant difference between participants based on their duration of experience in their profession (*p* ≤ 0.05), physicians working at hospitals with 20–50 ICU beds were 41.0% (OR: 0.59 (CI: 0.37–0.96)) less likely to be knowledgeable about IAH/ACS (*p* ≤ 0.05) Practice: Only 27.1% of the respondents had performed IAP measurement; 43.2% reported that they measure IAP for patients at risk for IAH, 41.4% reported that they do not measure IAP in medical patients, while around 37.0% reported that they do not measure IAP in surgical patients; more than half of them (62.8%) reported that they do not routinely measure IAP Management: The use of inotropes or vasopressors was the intervention that was most frequently reported (13.5%) in the treatment of ACS and IAH; 47.0% of those stated that they would never request or conduct surgical decompression on a patient with ACS, while nearly 44.0% of those who conduct/request surgical decompression in ACS patients based their choice on the severity of organ dysfunction. Barriers for IAP measurement: do not know how to measure IAP (42.5%), do not treat any patients with IAH (36.5%), rely on clinical/physical examination and assessment (25.4%), do not know how to interpret IAP (12.7%).
Wise et al. 2019 [12]	Africa	A cross-sectional survey. Method: an online survey using a self-made questionnaire with 53 questions	To determine the impact of the 2013 WSACS IAH/ACS consensus definitions and clinical management guidelines on IAH/ACS clinical awareness and management	A convenience sample of 559 healthcare professionals mainly included physicians (84.0%), nurses (5.4%) working in critical care units: mixed ICUs (87.3%; *n* = 448); surgical ICUs (7.8%; *n* = 40);medical ICUs(1.2%; *n* = 6), paediatric ICUs(1.8%; *n* = 9), trauma ICUs(1.4%; *n* = 7), and other ICUs (0.6%; *n* = 3)	Knowledge: the knowledge scores was low (onlly 48.0% of questions answered correctly), only 56.6% (313/559) knew the correct definition of IAH, while 53.0%(293/559) knew the correct definition of ACS; for paediatric population, the accuracy of answers regarding at which IAP level ACS occurs is very low. Practice: A considerable proportion of respondents (18%) never measure IAP, while 23.8% would measure IAP routinely and 28.6% would sometimes take readings; IAP was measured most frequently via the bladder (73.8%). Management: Treatment interventions of IAH/ACS were mainly achieved through diuretics (49.2%), inotropes (38.6%), decompressive laparotomy (37.0%), paracentesis (36.5%), and fluids/ blood products (24.2%). A decompressive laparotomy mainly depends on the degree of organ dysfunction (59.3%; *n* = 337) and the evolution of this dysfunction (51.9%; *n* = 295). Barriers for IAP measurement: reliance on physical examination (39%; *n* = 38), a lack of knowledge (15.3%; *n* = 15), a perceived lack of patients with IAH (13.3%; *n* = 13), or due to expense (5.1%; *n* = 5).
Strang et al. 2017 [27]	Netherlands	A cross-sectional study Method: A structured questionnaire based on literature and expert consensus was developed with 29 questions	To determine the current state of awareness, knowledge and use of evidence-based medicine regarding IAH and ACS among surgeons	A convenience sample of 60 surgeons from ICUs of different hospitals	Knowledge: Most respondents were not familiar with the WSACS guidelines for the definition and treatment of ACS. Forty-two (70%) respondents claimed to know the definition of IAH as set by the WSACS, whice thirty-one (52%) knew the definition of ACS as proposed by the WSACS Practice: only 16 (27%) respondents actually use the WSACS guidelines in daily practice; IAP measurements were performed in 58/60 (96%) of the hospitals; IAP was measured most frequently via the bladder (98.0%); a quarter of respondents (*N* = 14; 25%) measures IAP three times daily on average; Forty-nine (88%) respondents wait with measuring of IAP until there is a clear suspicion for ACS Management: Most respondents (*N* = 33; 55%) were not familiar with the WSACS guidelines for the treatment of ACS,7 (45%) were familiar with the guidelines, but only 16 (27%) actually use them in daily practice; the majority of respondents considered diuretics (*N* = 38; 63%) and laparotomy (*N* = 33; 55%) were very useful or fairly useful, while thirty-five (58%) respondents considered that IAH is only a symptom and as such needs no treatment; nearly all respondents (*N* = 59; 98%) believed that open abdomen management improves patient outcomes, many (*N* = 46; 77%) confirm the high complications rate of this treatment.
Zhang et al. 2016 [28]	China	A cross-sectional survey. Method: using a self-made questionnaire with 12 questions	To explore the physicians’ awareness of the 2013 World Society of Abdominal Compartment Syndrome (WSACS) guidelines in Chinese ICUs.	A convenience sample of 37 physicians (67.6%) and nurses (32.4%) in four intensive care units	Knowledge: there was a low awareness of the 2013 WSACS guidelines, especially in the area of IAH related risk factors, diagnostic threshold value of IAH, intravesical pressure measuring methods and indicators; the “correct” rate of each question was relatively low, the overall knowledge scores rate of the four intensive care units is only 30%. Only 13.5%(5/37) knew the correct definition of IAH, while 43.2%(16/37) knew the correct definition of ACS.
Wise et al. 2015 [16]	Africa	A cross-sectional survey. Method: an interactive online survey with 53 questions created by the WSACS executive committee.	To access the awareness, knowledge and management of IAH/ ACS of all members of the WSACS, the European Society of Intensive Care Medicine, the Society of Critical Care Medicine and participants of the 3rd World Congress on Abdominal Compartment Syndrome meeting	A convenience sample of 2,244 healthcare workers mainly included physicians (63.9%), nurses (10.6%) working in a mixed medical/trauma/surgical/ paediatric ICU	Awareness:1,909 (85.6%) respondents claimed to be familiar with IAP and IAH while 1,903 (98.8%) were familiar with ACS. Knowledge: knowledge of the consensus definitions on IAH and ACS was even lower at 31.0%, only 32.2% adult intensivists knew the correct definition of IAH/ACS. Adult intensivists had the highest score (43.4 ± 14.6) vs. nurses (41.6 ± 17.4) and others (39.7 ± 13.2) (*P* < 0.05); knowledge of the WSACS was low, with an overall figure of 42.5%; respondents who were aware of the WSACS had a better score compared to those who were not (49.6% vs. 38.6%, *P* < 0.001). Practice: 4% of respondents did not measure IAP; IAP was measured most frequently via the bladder (91.9%), but the minority can accurately measure IAP according to the current guidelines, including correct volumes of saline, reading timing; the frequency of IAP monitoring was variable. Management: Surgical decompression was frequently used to treat IAH/ACS, whereas medical management was only attempted by about half of the respondents; decisions to decompress the abdomen were predominantly based on the severity of IAP elevation and presence of organ dysfunction (74.4%). Barriers for IAP measurement: A lack of knowledge about measurement techniques and how to interpret its value
Da Silva et al. 2012 [32]	Brazil	A cross-sectional study Method: using a self-made questionnaire with 13 multiple choice questions	To evaluate the intensivist’s knowledge on ACS	A convenience sample of 32 intensivists in seven ICU	Knowledge: with respect to the consensus definitions by the World Society of the Abdominal Compartment Syndrome, only 21.8% claimed to be aware of the definitions of IAH and ACS; 34.4% of intensivists reported they have ignored the main adverse effects of IAH. Practice: Most respondents (87.8%) stated that IAP measurement should be routine at ICUs, but only 34% of intensivists had measured IAP; IAP was measured most frequently via the bladder (91.0%); As for the frequency of IAP measurement, 37% said that should be based on clinical data from patient, while 63% said there should be a standardized frequency (18% worked with intervals of 8 h, 18% of 12 h, and 27% of 4 h between measurements). Management: 32% of intensivists stated that they have never examined patients at risk for IAH/ACS at the units where they worked. Barriers for IAP measurement: lack of knowledge of the measurement technique (40%), lack of knowledge on how to interpret it (24%), waste of time (1%).
Zhou et al. 2011 [33]	China	A cross-sectional study Method: using a self-made questionnaire with 20 items	To clarify the current understanding and clinical management of IAH/ACS among intensive care physicians in tertiary Chinese hospitals	A convenience sample of 108 intensive care physicians (75.0% in combined medical-surgical ICUs, 11.1% in a medical ICU, 8.3% in a surgical ICU, 2.8% for neurological ICU, and 2.8% for “other”) participanting the 3rd chinese national critical care conference	Knowledge: there was low knowledge on the correct volume of saline that should be instilled (12,16.0%) and the zero reference points for the measurement (9,12.0%) Practice: one third (30.6%, *n* = 33) of ICUs never measured IAP in their intensive care practice, among those ICUs that measured IAP, the majority 88.0% (*n* = 66) measured IAP when there was a clinical suspicion of IAH/ACS, 8.0% (*n* = 6) measured IAP on patients after emergency laparotomy, whereas only 4.0% (*n* = 3) measured IAP routinely in those who underwent massive fluid resuscitation; IAP was measured most frequently via the bladder (100.0%); Among those who measured IAP, only 29.3% (*n* = 22) measured either often or routinely; Management: Nearly three quarters of respondents preferred diuresis and dialysis (72% for both), followed by paracentesis (68%) and decompression laparotomy (56%), 28%, 28%, and 12%, respectively mentioned “pressors/ inotropes”, “fluid/ blood products”, and “sedation and neuromuscular blockade agents” for the treatment of ACS. Barriers for IAP measurement: “Do not know how to interpret the results obtained” (36.4%), “Never admit any patients with IAH”(27.3%)
Biancofiore et al. 2008 [35]	Italy	A cross-sectional study Method: using a self-made questionnaire with 5 items	To investigate the knowledge, recognition and management of IAP and IAH in Italian Intensive Care Units	A convenience sample of 77 Intensive Care Unit lead physician of italian hospitals	Knowledge: the knowledge of the definition of IAH was low, only 19.5% (10/51) can correctly define IAH/ACS. Practice: 51 (66.3%) ICUs measured IAP in their intensive care practice; in units where IAP was measured, the only method used was the urinary bladder method; most ICUs (20,39.2%) measured IAP when necessary, and the most frequent interval of measurement was once every 4 h; the most frequent indications for measuring IAPwere the presence of risk factors for IAH (64.7%) and a previous urgent surgery (21.5%) Management: when faced a patient with very high IAP (> 25 mmHg), the possible abdominal decompression was considered in 64.7% of the cases, whereas the concomitant presence of signs of organ dysfunction prompted surgical consultation in another 33.3%. Barriers for IAP measurement: “do not have the materials for measuring IAP”(34.7%); “do not know how to measure IAP”(23.0%); “never have to deal with patients with IAH”(15.4%); “do not know how to interpret IAP data”(11.0%); “it is a waste of time” (7.7%); “the data is of no use”(7.7%)
**Adult intensive care nurses**					
Chen et al. 2023 [22]	China	A cross-sectional study Method: using a self-made questionnaire with 45 items	To investigate the management and knowledge of IAP monitoring among intensive care unit nurses in secondary and tertiary general hospitals in china	A convenience sample of 1049 intensive care unit nurses	Knowledge: 42.7% (448/1049) of intensive care nurses knew the correct definition of IAH, while 45.3% (475/1049) knew the correct definition of ACS.The score of knowledge about IAP monitoring was low, with a score rate less than 40% (7/19), especially items related to the definition of IAH, measures for sustained elevation of IAP and IAP measurement. Practice: A total of 650 nurses (62.0%) had performed IAP monitoring; IAP was measured most frequently via the bladder (612, 94.2%); among nurses who have not performed IAP measurement, 62.7% have not received IAP related trainings; most of the nurses had an incompetent level of IAP measurement practice especially in the area of reference zero, volumes of saline, waiting time before the reading. Barriers for IAP measurement: lack of IAP monitoring related trainings (62.7%), the patients had no indications for IAP monitoring (45.9%), and insufficient understanding of the relevant evidence supporting IAP monitoring (45.4%).
Liu et al. 2023 [21]	China	A cross-sectional survey. Method: an online survey using a self-made questionnaire with 45 questions	To access the current situation of practice and cognition of ICU nurses on IAP monitoring in China	A convenience sample of 627 ICU nurses	Knowledge: Only 44.3% (278/627) knew the correct definition of IAH, while 44.2% (277/627) knew the correct definition of ACS; only 5 questions had a correct answer rate of ≥ 50%; nurses aged ≥ 41 years, nursing experience ≥ 21 years, departments with low bed-to-nurse ratio and participating in trainings related to IAP monitoring have higher knowledge scores. Practice: The implementation rate of IAP monitoring was relatively low (73.4%), IAP was measured most frequently via the bladder (96.7%); only 111 (24.9%) nurses chose the intersection of the midaxillary line and the iliac crest as the reference zero point measurement, 221(49.7%) nurses not took an reading at the end of expiration, only 274(61.6%) nurses chose volumes of saline that meets the recommended guidelines. Barriers for IAP measurement: the main reasons were that nurses had not received IAP monitoring related training (61.1%), the patients had no indications for IAP monitoring (47.31%), and insufficient understanding of the relevant evidence supporting IAP monitoring (46.7%).
Reyad et al. 2022 [26]	Africa	A cross-sectional study Method: using the self-made knowledge assessment questionnaire with 18 items, and the observational checklist adapted from Tayebi et al. with 10 items	To assess nurses’ knowledge and practice regarding IAP measurement and ACS prevention	A convenience sample of 60 nurses of intensive care units, cardiac care units and emergency unit	Knowledge: half (50%) of the intensive care nurses knew the definition of IAH, and 33.3% of the intensive care nurses were aware of the definition of ACS; 80% of the nurses had an unsatisfactory level of knowledge, epecially items related to the definition of IAP/IAH/ ASC, IAP measurement, and complications of ASC, while only 20% had a satisfactory level of knowledge. Practice: More than two thirds (71.7%) of the nurses had an incompetent level of practice.
Hunt et al. 2017 [15]	Australia	A cross-sectional study Method: an on-line Survey using a self-made questionnaire with 19 items	To assess the knowledge of critical care nurses about current IAH and ACS practice guidelines, measurement techniques, predictors for the development of IAH and ACS and identify barriers in recognizing IAH, ACS and measuring IAP	A convenience sample of 86 registered nurses employed in critical care units	Knowledge: Most of critical care nurses were able to identify some obvious causes of IAH (e.g. abdominal surgery and major trauma), while less than 20% were able to recognize less apparent indices of risk (e.g. acidosis, hypothermia); Most of critical care nurses were able to identify some more apparent clinical manifestations of ACS, while only a small portion of participants were able to recognize less apparent clinical signs of ACS Practice: over three-quarters (77.6%, *n* = 66) of participants monitored for IAH and ACS within their workplace and the same percentage had a policy for IAH and ACS monitoring and management. Barriers for IAP measurement: The lack of education related to IAP monitoring (44.2%), low level of nursing staff support (28%), low level of surgeon support (27%), low evidence to support the practice (27%) and lack of confidence in undertaking the assessment (27%). Facilitators to IAP measurement: Education and support for IAP monitoring (73%), clear and accessible protocols and policies (65%), evidence based guidelines (61%) and intensivist endorsement of practice (53%).
Wise et al. 2015 [16]	Africa	A cross-sectional survey. Method: an interactive online survey with 53 questions created by the WSACS executive committee.	To access the awareness, knowledge and management of IAH/ ACS of all members of the WSACS, the European Society of Intensive Care Medicine, the Society of Critical Care Medicine and participants of the 3rd World Congress on Abdominal Compartment Syndrome meeting	A convenience sample of 2,244 healthcare workers mainly included physicians (63.9%), nurses (10.6%) working in a mixed medical/trauma/surgical/ paediatric ICU	Knowledge: only 27.4% adult intensive care nurses knew the correct definition of IAH/ACS; knowledge of the WSACS was low, with an overall figure of 34.4%. Practice: 4% of respondents did not measure IAP; IAP was measured most frequently via the bladder (91.9%), but the minority can accurately measure IAP according to the current guidelines, including correct volumes of saline, reading timing; the frequency of IAP monitoring was variable.

### Awareness of IAH/ACS

Eight studies explored awareness of IAH/ACS among intensive care providers [16, 23–25, 29–31, 34]. Two surveys revealed that 73.7–98.8% of adult intensivists reported being familiar with IAH/ACS. Qutob et al. [24] reported that among ICU physicians familiar with ACS, approximately one-third (28.2%) had encountered 1 to 5 cases of ACS in the past year. The data indicated that pediatric intensive care providers had a lower awareness of IAH/ACS compared to adult intensivists. According to five surveys [23, 25, 29, 30, 34], awareness of IAH/ACS among pediatric intensivists ranged from 44.0 to 97.0%, compared to 50.0–95.0% among pediatric intensive care nurses in three surveys [29, 31, 34]. IAH and ACS were not uncommon in the pediatric population. In Kaussen’s study [30], nearly half of pediatric intensivists (56/123) reported encountering IAH/ACS in daily practice, with 36.0% (44/124) noting at least one case of IAH and 26.0% (32/124) noting at least one case of ACS. Similarly, Wiegandt et al. [23] reported an increase in the percentage of PICUs diagnosing at least one case of ACS, from 25.0 to 35.0%. Awareness of IAH/ACS appeared to be correlated with the length of ICU service, as the proportion of medical staff aware of IAH/ACS increased with more experience working in the ICU [29, 31].

### Knowledge of IAH/ACS

All nineteen studies assessed the knowledge of the consensus definition of IAH/ACS among intensive care providers. Data from seven surveys demonstrated that 13.5–70.0% of adult intensivists could correctly define IAH/ACS according to the WSACS consensus definition, with an increasing trend observed since the 2008 survey results [12, 16, 24, 27, 28, 32, 35]. Similar levels of knowledge and trends were also reported among adult intensive care nurses (27.4–50.0%) in five surveys [15, 16, 21, 22, 26]. However, the reported knowledge of IAH/ACS among pediatric intensive care providers appeared to be lower than that of adults. Data from five surveys showed that only 4.0–58.0% of pediatric intensivists knew the correct definition of IAH/ACS [23, 25, 29, 30, 34]. In four surveys, the knowledge rate regarding the definition of IAH/ACS was below 20.0% [23, 25, 29, 30], and showed a significant downward trend since the 2007 survey results. A nationwide cross-sectional survey by Kaussen et al. [30] found that only 16.5% (21/127) of pediatric intensivists knew the correct consensus definitions of IAH/ACS for critically ill children. Data from four surveys also showed a low level of knowledge regarding the definition of IAH/ACS among pediatric intensive care nurses (13.2–30.0%) [20, 29, 31, 34]. Studies conducted in China indicated that intensive care nurses who participated in training related to IAP measurement had better knowledge scores than those who had not received such training (*P* < 0.05) [20]. Table 2. summarizes the knowledge and awareness of IAH/ACS among intensive care providers.

**Table 2:** Knowledge and awareness of IAH/ACS

Speciality	Study	Knowledge of IAH/ACS	Awareness of IAH/ACS
**Pediatric intensive care nurses**	Li et al. 2024 [20]	30.0% pediatric intensive care nurses correctly defined IAH/ACS in children	/
	Liang et al. 2015 [29]	25.0% (1/30) knew the correct definition of ACS	50% pediatric intensive care nurses heard of ACS
	Newcombe et al. 2012 [31]	13.2% respondents correctly defined ACS in children	87.8% pediatric intensive care nurses aware of ACS
	Ejike et al. 2010 [34]	24.7% pediatric intensive care nurses correctly defined ACS in children	95.0% pediatric intensive care nurses aware of ACS
**Pediatric intensivists**	Wiegandt et al. 2023 [23]	4% pediatric intensivists in 2010 and 6% in 2016 knew the correct definition of IAH, while 18% in 2010 and 58% in 2016 knew the correct definition of ACS	More physicians reported that IAH and ACS play a role in clinical practice compared to 2010(44%), 2016(55%)
	Rezeni et al. 2022 [25]	10% pediatric intensivists correctly defined ACS as per the WSACS consensus definition	93.7% (*n* = 74/79) pediatric intensivists were aware of ACS
	Liang et al. 2015 [29]	8.7% pediatric intensivists knew the correct definition of ACS	74.7% pediatric intensivists heard of ACS
	Kaussen et al. 2012 [30]	No more than 3.9% (5/127) of respondents knew correct IAH definition, while no more than 16.5% (21/127) knew correct definition of ACS	45.5% of pediatric intensivists that responded stated IAH and ACS are present in everyday clinical practice
	Ejike et al. 2010 [34]	55.8% pediatric intensivists correctly defined ACS in children	97.0% of pediatric intensivistss aware of ACS
**Adult intensivists**	Biancofiore et al. 2008 [35]	19.5% (10/51) adult intensivists can correctly define IAH/ACS	
	Da Silva et al. 2012 [32]	21.8% adult intensivists knew the consensus definitions of IAH/ ACS	/
	Wise et al. 2015 [16]	32.2% adult intensivists have knowledge of the consensus definitions on IAH/ACS	85.6% adult intensivists claimed to be familiar with IAH while 98.8% were familiar with ACS
	Zhang et al. 2016 [28]	13.5% (5/37) knew the correct definition of IAH, while 43.2%(16/37) knew the correct definition of ACS	/
	Strang et al. 2017 [27]	Forty-two (70%) respondents claimed to know the definition of IAH, whice thirty-one (52%) knew the definition of ACS	/
	Wise et al. 2019 [12]	56.6% (313/559) knew the correct definition of IAH, while 53.0% (293/559) knew the correct definition of ACS	/
	Qutob et al. 2022 [24]	33.8% knew the correct definition of IAH, while 39.8% knew the correct definition of ACS	73.7% adult intensivists claimed to be familiar with ACS
**Adult intensive care nurses**	Reyad et al. 2022 [26]	50% of the studied nurses knew the definition of IAH, more than half (33.3%) of the studied nurses knew the definition of ACS	/
	Chen et al. 2023 [22]	42.7% (448/1049) knew the correct definition of IAH, while 45.3% (475/1049) knew the correct definition of ACS	/
	Liu et al. 2023 [21]	Only 44.3% (278/627) knew the correct definition of IAH, while 44.2% (277/627) knew the correct definition of ACS.	/
	Wise et al. 2015 [16]	27.4% adult intensive care nurses knew the correct definition of IAH and ACS	/

### IAP measurement practice

#### The implementation of IAP measurement

All surveys indicated that IAP measurement has not been effectively implemented in clinical practice, with 18.0–73.0% of intensive care providers never measuring IAP, particularly for routine measurements [12, 16, 20, 21, 23–25, 27]. Five surveys indicated that 12.4–88.0% of intensive care providers only performed IAP measurements for patients at risk of IAH or with a clear suspicion of ACS [24, 25, 30, 33, 35]. Kaussen et al. [30] found that 17.0% of pediatric intensivists only measured IAP in cases of organ dysfunction and failure. IAP was most frequently measured via the bladder [12, 16, 21, 22, 27, 29–35]; however, several surveys revealed that only a minority of participants could accurately measure IAP in accordance with current guidelines, including correct saline volumes, reference zero, and timing of readings [12, 16, 20, 21, 25, 26]. The frequency of IAP monitoring varied. Rezeni et al. [25] discovered that 28% of pediatric intensivists measured IAP every 6 h, 28% every 12 h, and 12% only when clinically indicated, with none performing continuous monitoring. In the study by Da Silva [32], 37.0% of intensivists believed IAP monitoring frequency should be based on clinical data from patients, while 63.0% favored a standardized frequency. Furthermore, 18.0% measured IAP every 8 h, 18.0% every 12 h, and 27.0% every 4 h.

#### Barriers and facilitators for IAP measurement

According to twelve survey results, the implementation of IAP measurements is influenced by factors such as insufficient knowledge of IAP measurement and result interpretation [12, 15, 16, 20–22, 24, 32, 33, 35], lack of familiarity with IAP measurement techniques [16, 20–22, 24, 25, 32, 35], reliance on clinical or physical examinations [12, 24, 25, 30], limited experience with IAH patients [12, 24, 33, 35], the perception of wasted time [20, 32, 35], lack of necessary measurement equipment [30, 35], and challenges in identifying high-risk populations that require IAP measurements [20]. Difficulty identifying high-risk IAH populations is the main barrier to IAP measurement among pediatric intensive care nurses [20]. Conversely, reliance on physical examinations is a common barrier among pediatric intensivists [25, 30], while adult intensivists [12, 24, 26, 32–34] and adult intensive care nurses [15, 21, 22] frequently cited a lack of knowledge in measuring and interpreting IAP data as significant barriers. Two studies identified facilitators for IAP measurement. Hunt et al. [15] highlighted education and support as key facilitators among intensive care nurses, while Kaussen et al. [30] noted that simplified, standardized procedures and technical requirements enabled pediatric intensivists to measure IAP more frequently. Table 3. summarizes the barriers for IAP measurement, while Fig. 6, 7, 8, 9 show the frequency of mentioned barriers by intensive care providers.

**Table 3:** Barriers for IAP measurement

Speciality	Study	Barriers for IAP measurement
**Pediatric intensive care nurses**	Li et al. 2024 [20]	1. Difficulty in identifying the high-risk population of IAH (64.6%) 2. Did not know how to measure IAP (55.6%) 3. Unreasonable nurse patient ratio allocation (52.8%) 4. Do not konw how to interpret IAP data (43.4%) 5. It is unnecessary to measure IAP (10.7%)
**Pediatric intensivists**	Rezeni et al. 2022 [25]	1. Relied on their physical assessment (*n* = 14, 45%) 2. Did not know how to measure IAP (*n* = 10, 32%) 3. Did not think it was a frequent condition in paediatrics (*n* = 7, 22.5%)
	Kaussen et al. 2012 [30]	1. Clinical diagnosis (IAP measurement not necessary) (48%) 2. Lack of technical equipment (42%) 3. Lack of therapeutical consequence (11%) 4. Fear for invasiveness (9%)
**Adult intensivists**	Biancofiore et al. 2008 [34]	1. Lack of technical equipment for measuring IAP (34.7%) 2. Do not know how to measure IAP (23.0%) 3. Never have to deal with patients with IAH (15.4%) 4. Do not know how to interpret IAP data (11.0%) 5. It is a waste of time (7.7%) 6. The data is of no use (7.7%)
	Zhou et al. 2011 [33]	1. Do not know how to interpret the results obtained (36.4%) 2. Never admit any patients with IAH (27.3%)
	Da Silva et al. 2012 [32]	1. Lack of knowledge of the measurement technique (40%) 2. Lack of knowledge on how to interpret it (24%) 3. Waste of time (1%)
	Wise et al. 2015 [16]	1. A lack of knowledge about measurement techniques 2. Do not know how to interpret IAP data
	Wise et al. 2019 [12]	1. Reliance on physical examination (39%) 2. A lack of knowledge (15.3%) 3. A perceived lack of patients with IAH (13.3%) 4. Due to expense (5.1%)
	Qutob et al. 2022 [24]	1. Do not know how to measure IAP (42.5%) 2. Do not treat any patients with IAH (36.5%) 3. Rely on clinical/physical examination and assessment (25.4%) 4. Do not know how to interpret IAP (12.7%)
**Adult intensive care nurses**	Hunt et al. 2017 [15]	1. The lack of education related to IAP monitoring (44.2%) 2. Low level of nursing staff support (28%) 3. Low level of surgeon support (27%) 4. Low evidence to support the practice (27%) 5. Lack of confidence in undertaking the assessment (27%)
	Chen et al. 2023 [22]	1. Lack of IAP monitoring related trainings (62.7%) 2. The patients had no indications for IAP monitoring (45.9%) 3. Insufficient understanding of the relevant evidence supporting IAP monitoring (45.4%)
	Liu et al. 2023 [21]	1. Had not received IAP monitoring related training (61.1%) 2. The patients had no indications for IAP monitoring (47.31%) 3. Insufficient understanding of the relevant evidence supporting IAP monitoring (46.7%)

### Treatments for IAH and ACS

Only one survey has reported the adoption of the WSACS guidelines for the treatment of ACS among intensive care providers. Strang et al. [27] revealed that less than half of adult intensivists (45.0%) were acquainted with the WSACS guidelines for ACS treatment, with only a quarter of them (27.0%) incorporating them into their daily practice. Diuretics were the most frequently mentioned intervention for managing IAH/ACS, followed by the administration of vasopressors and inotropes, decompressive laparotomy, judicious administration of fluids and blood products, and the utilization of abdominal paracentesis [12, 16, 23–25, 27, 30–33, 35]. The inclination to employ decompressive laparotomy for treating ACS appears to differ among intensivists of various specialties. Based on six survey findings, 37.0–66.3% of adult intensivists would opt for decompressive laparotomy in ACS cases [12, 16, 24, 27, 33, 35]. In contrast, this was the less likely reported therapeutic option among pediatric intensivists in three surveys [23, 25, 30]. Wise et al. [16] found that surgical intensivists were the group most inclined to conduct decompressive laparotomy for treating ACS in their patients, with pediatric intensivists being the least likely among various disciplines. Kaussen et al. [30] highlighted that the decision to proceed with decompressive laparotomy is significantly influenced by the patient’s age and the presence of organ dysfunction. More invasive therapy options will not be considered until IAP ≥ 20 mmHg (as long as organ dysfunction remains absent). The criteria for deciding to perform abdominal decompression predominantly included the degree of organ dysfunction [12, 16, 24, 35], the evolution of organ dysfunction [12], and the severity of IAP elevation [16]. Table 4. summarizes the treatments for IAH and ACS.

**Table 4:** Treatments for IAH and ACS

Speciality	Study	Treatments for IAH and ACS
**Pediatric intensivists**	Wiegandt et al. 2023 [23]	36% respondents using decompressive laparotomies to reduce IAP; respondents considered that the survival rate of ACS was higher if the patient was treated surgically.
	Rezeni et al. 2022 [25]	Diuresis and sedationanalgesia were the most frequently used therapeutic approaches to treat IAH and ACS, while decompression laparotomy was the less likely reported therapeutic option.
	Kaussen et al. 2012 [30]	Unlike older children, the likelihood of young pediatric patients undergoing more invasive therapy options earlier appears to be inversely related to their age; more invasive therapy options will not being used until IAP ≥ 20 mmHg as long as organ dysfunction remains absent.
**Adult intensivists**	Qutob et al. 2022 [24]	The use of inotropes or vasopressors was the intervention that was most frequently reported (13.5%) in the treatment of ACS and IAH; 47.0% of those stated that they would never request or conduct surgical decompression on a patient with ACS, while nearly 44.0% of those who conduct/request surgical decompression in ACS patients based their choice on the severity of organ dysfunction.
	Wise et al. 2019 [12]	1. Treatment interventions of IAH/ACS were mainly achieved through diuretics (49.2%), inotropes (38.6%), decompressive laparotomy (37.0%), paracentesis (36.5%), and fluids/ blood products (24.2%). 2. A decompressive laparotomy mainly depends on the degree of organ dysfunction (59.3%; *n* = 337) and the evolution of this dysfunction (51.9%; *n* = 295). 3. Most respondents would perform/request a decompressive laparotomy in cases of ACS, with 64.4% of respondents indicated that decompression would be preferred in selected cases only
	Strang et al. 2017 [27]	1. Most respondents (*n* = 33; 55%) were not familiar with the WSACS guidelines for the treatment of ACS,7 (45%) were familiar with the guidelines, but only 16 (27%) actually use them in daily practice; 2. The majority of respondents considered diuretics (*n* = 38; 63%) and laparotomy (*n* = 33; 55%) were very useful or fairly useful, while thirty-five (58%) respondents considered that IAH is only a symptom and as such needs no treatment; 3. Nearly all respondents (*n* = 59; 98%) believed that open abdomen management improves patient outcomes, many (*n* = 46; 77%) confirm the high complications rate of this treatment.
	Wise et al. 2015 [16]	1. Surgical decompression was frequently used to treat IAH/ACS, whereas medical management was only attempted by about half of the respondents; 2. Decisions to decompress the abdomen were predominantly based on the severity of IAP elevation and presence of organ dysfunction (74.4%)
	Zhou et al. 2011 [33]	Nearly three quarters of respondents preferred diuresis and dialysis (72% for both), followed by paracentesis (68%) and decompression laparotomy (56%), 28%, 28%, and 12%, respectively mentioned “pressors/ inotropes”, “fluid/ blood products”, and “sedation and neuromuscular blockade agents” for the treatment of ACS
	Biancofiore et al. 2008 [35]	When faced a patient with very high IAP (> 25 mmHg), the possible abdominal decompression was considered in 64.7% of the cases, whereas the concomitant presence of signs of organ dysfunction prompted surgical consultation in another 33.3%.

## Discussion

This scoping review thoroughly assessed intensive care providers’ awareness, knowledge, and practices regarding IAP/IAH/ACS across 19 cross-sectional studies involving 7363 participants from 9 countries and regions. Although there has been progress in awareness and knowledge regarding the WSACS consensus and guidelines since their publication in 2007, it is still insufficient to effectively implement these recommendations in the management of IAH/ACS.

Compared to the baseline survey on IAH/ACS awareness, a growing proportion of participants reported familiarity with IAH/ACS over the years. Among the participants, adult intensivists demonstrated the highest level of awareness of IAH/ACS, followed by pediatric intensive care nurses, and then pediatric intensivists. Despite an improvement in the awareness and knowledge of IAH/ACS, on average, only about two-fifths of adult intensivists, less than one-third of pediatric intensivists and adult intensive care nurses, as well as no more than one-quarter of pediatric intensive care nurses were able to correctly define IAH/ACS. IAH and ACS were not uncommon in the pediatric population [36, 37]. However, in certain surveys, only a small minority of pediatric intensive care providers correctly recognized that the development of new organ dysfunction/failure, along with elevated IAP, constitutes the definition of ACS. This indicates an ongoing confusion among pediatric intensive care providers regarding the accurate definition of ACS, which may be partly attributed to the diverse range of patient ages and sizes encountered daily by pediatric intensive care providers [38]. A lack of comprehensive knowledge could impact the classification and diagnosis of patients with IAH/ACS, consequently hindering the timely identification and intervention [39]. Regardless of the underlying reason for this limited knowledge, continuous knowledge updating and regular competency assessment are crucial in improving professional proficiency [40].

According to the WSACS guidelines, it is recommended to measure IAP in critically ill patients when any risk factors for IAH/ACS are present [11]. Identifying exactly which patients are at increased risk of developing IAH/ACS is important to avoid unnecessary IAP measurement. Further, it is crucial to prevent progression to ACS through early identification and intervention in those with IAH, thereby preventing irreversible organ damage or mortality [41]. The risk factors for IAH/ACS have been described, which can be largely divided into three categories: decreased abdominal compliance, increased intra-abdominal volume, and a combination of both [11]. However, these risk factors were predominantly based on expert opinion or pathophysiology and were supported by only weak evidence, making their application at the bedside challenging. The meta-analysis conducted by Holodinsky et al. revealed that sepsis, obesity, ileus, abdominal surgery, and fluid resuscitation were identified as risk factors for IAH among mixed ICU patients. In trauma and surgical patients, crystalloid resuscitation was the most common risk factor for ACS, while higher APACHE II/Glasgow-Imrie scores and elevated serum creatinine were associated with ACS in severe acute pancreatitis cases [42]. To precisely identify high-risk patients for IAH, it may be critical to establish collaborative efforts among various critical care specialties to pinpoint patients in the unit who are at high risk and to monitor their IAP.

Intermittent IAP measurement via the bladder, initially outlined by Kron et al. in 1984, stands as the most widely embraced method due to its simplicity, reliability, user-friendliness, cost-effectiveness, and minimal invasiveness [43]. This technique was subsequently refined by Cheatham and Malbrain into a closed system repeated measurement technique using a pressure transducer [44]. IAP measurements can also be performed by measuring the height of the urine column with a Foley manometer [43]. Intermittent IAP measurements via the bladder every 4 to 6 h in symptomatic patients or those with a high clinical suspicion of developing ACS are widely accepted as routine practice. However, continuous measurement may be preferable when patients with ACS require emergency abdominal decompression. Currently, different techniques are available for continuous measurement and estimation of IAP [45, 46]. Continuous measurement is best for long-term, fully automated monitoring of IAP, as it is less prone to errors and proves to be the most cost-effective option when utilized over an extended period [47]. Considering the frequent requests for IAP measurements in ICU patients, it is essential to identify an accurate, simple, quick, and less invasive method. Numerous noninvasive techniques have been developed, including abdominal wall tension measurement, wireless motility capsule, and near-infrared spectroscopy; these may have substantial potential to replace traditional IAP measurement technologies [48].

IAH is a continuum from (often) asymptomatic elevation of IAP to an immediately life-threatening situation (fulminant ACS). Treatment of IAH should be guided by both the underlying causes leading to IAH and the degree of organ dysfunction. Numerous medical and minimally invasive therapies have been proposed or studied, offering potential benefits for individuals afflicted by IAH [49]. These therapies aim to improve abdominal wall compliance, evacuate intraluminal and extraluminal contents, and correct fluid balance to prevent further deterioration [50]. Potential approaches or techniques encompass sedation and analgesia, diuretics and continuous renal replacement therapies, neuromuscular blockade, fluid resuscitation strategies, nasogastric/colonic decompression, and percutaneous catheter drainage. According to current knowledge, decompressive laparotomy should be reserved for cases in which noninvasive medical interventions fail to reduce IAP and organ failure persists [11]. This is due to the considerable consequences, as evidenced by high reported mortality rates in both adults (49.7%) and children (60.8%), even following decompression [51]. Nevertheless, in cases of rapid progression to ACS, especially in primary ACS, decompressive laparotomy should be performed promptly since it is the definitive treatment to reduce the risk of mortality from ACS [50].

### Limitations

This scoping review had several limitations. For example, some studies may have been missed if they appeared in additional databases that were not searched in this review. Moreover, the predominant focus of studies in Western countries has resulted in a lack of diverse cultural representation in the reviews. Furthermore, the questionnaires utilized in the majority of studies lacked standardization and validation, as they were non-validated researcher-designed instruments, thus introducing levels of subjectivity. No quality assessment of the included studies was conducted when evaluating the features and methodology of the scoping review [18]. Nevertheless, a scoping review aims to present a general map of the research on a general topic to inform readers and to clarify the needs for further research, which has been achieved in our study. Finally, most included studies were cross-sectional and relied on self-reported surveys to assess the awareness, knowledge, and practices of intensive care providers. Although self-reported surveys on awareness, knowledge, and practices are valuable, it is important to note that self-reported practices may not always align with actual clinical practices.

## Conclusions

Although awareness and knowledge of IAP/IAH/ACS among intensive care providers have improved since the publication of the 2007 WSACS consensus, their understanding of the definitions of IAH/ACS remains insufficient, particularly among pediatric intensive care providers. Survey data indicate that the implementation of IAP measurement in clinical practice has been suboptimal, with a notable lack of knowledge about measuring and interpreting IAP data identified as a key barrier. To address this gap, urgent efforts are needed to improve adherence to WSACS guidelines and standardise IAP monitoring in routine practice.

## Data Availability

The datasets used and/or analysed during the current study are available from the corresponding author on reasonable request.
